# Human-centered design and maternity care: is this a possible interplay?—a systematic review

**DOI:** 10.1186/s12884-024-07119-1

**Published:** 2025-03-08

**Authors:** Filipa Landeiro, Mónica Silva, Carolina Veiga e Moura, Catarina Martins, Paula Miller, Sandrina Ferraz, Andréa Paula de Azevedo, Sara Gancho, Luís Rocha, Rui Patrício, Inês Nunes

**Affiliations:** 1 Universidade Europeia, IADE, Faculdade de Design, Tecnologia e Comunicação, UNIDCOM/IADE, Unidade de Investigação em Design e Comunicação, Av. D. Carlos I, 4, 1200-649 Lisboa, Portugal; 2 Universidade Europeia, IADE, Faculdade de Design, Tecnologia e Comunicação, Av. D. Carlos I, 4, 1200-649 Lisboa, Portugal; 3 Departamento da Mulher e Medicina Reprodutiva, Unidade Local de Saúde de Santo António, Porto, Portugal; 4 Escola Superior de Enfermagem do Porto, ESEP, Porto, Portugal; 5 Instituto de Puericultura e Pediatria Martagão Gesteira, Universidade Federal do Rio de Janeiro, Rio de Janeiro, Brasil; 6 Consultant, Lisbon, Portugal; 7 CINTESIS - Centro de Investigação Em Tecnologias e Serviços de Saúde, University of Porto, Porto, Portugal; 8 GOVCOPP and DEGEIT, Department of Economics, Management, Industrial Engineering and Tourism, University of Aveiro, Aveiro, Portugal

**Keywords:** Maternity care, Childbirth care, Humanization, Co-creation, Design, Healthcare

## Abstract

This paper argues that putting women at the center of care requires the right balance between adequate clinical care and human-centered design (HCD) approaches. Enhancing their experience during the maternity journey would make it possible to address societal challenges and effectively achieve the humanization of maternity care. Thus, the aim is to investigate the interplay between human-centered design and maternity care through a literature review. MEDLINE (Pubmed), CINAHL (EBSCO), Web of Science, and Scopus databases were searched, and twenty-one papers were selected as primary studies according to predefined inclusion criteria and as per agreement of the authors, either from design/social sciences or clinical backgrounds. Studies from eight countries targeting prenatal, childbirth, and/or postnatal care were reviewed, including healthcare professionals and pregnant women as participants. A systematic approach was followed for the papers, and specific attention was paid to socioeconomic and racial issues. The last phase included prototype testing, which involved digital resources development. Creating solutions for the mainstay problems throughout HCD is a helpful tool in surpassing systems’ problems and disadvantages, allowing for identifying and accurately targeting healthcare system gaps and maternity care opportunities to achieve a positive and humanized journey.

## Background

In the context of maternity care, humanization has emerged as a response to the patriarchal and biomedical model of childbirth, which heavily relies on medical interventions that may not always be necessary or evidence-based, leading to iatrogenic morbidity and reduced humanization of care for women.

Initially, the term humanization in maternity care aimed to promote less medicalized childbirths. [1]

Humanization in the field of maternity care has faced considerable criticism for being associated solely with low-risk, natural, or normal childbirth. Its meaning has expanded beyond being a mere opposition to the biomedical model of childbirth to include a range of practices that deliver personalized care to women. This approach is founded on evidence-based practices prioritizing safety while providing unique requirements to each woman. [1] Regardless of its definition, women in the perinatal stage have consistently requested care that provides confidence, trust, respect, privacy, a decision-making process, and control. [2]

Studies indicate that women aspire to have a good pregnancy experience that includes “sustaining physical and sociocultural normalcy, ensuring the health and well-being of both mother and baby (including prevention and treatment of risks, illnesses, and mortality), achieving a successful transition to labor and childbirth, and attaining positive motherhood (self-esteem, competence, and autonomy)”. [3] A more defined comprehension of the humanization concept concerning childbirth is crucial in different clinical settings.

While woman-centered care has long been a central paradigm in maternity care, focusing on women's clinical and emotional needs, human-centered design offers a complementary and transformative perspective, addressing clinical outcomes and the broader, non-clinical aspects of the care experience. This approach ensures that the design of services and systems responds holistically to women's lived realities, incorporating their voices at every stage of problem-solving and solution development.

Healthcare systems must constantly evolve to meet the needs of patients and providers [4–6]. While the humanization of maternity care has gained traction globally, there still needs to be more systematic approaches to achieve this aim. Remarkably, there needs to be more research on whether structured methodologies like HCD could address key challenges, such as mismatched interventions or unresponsive services. Incorporating this perspective highlights how the design may represent a transformative opportunity in this field.

Applying human-centered design (HCD) to healthcare could enhance innovation, efficiency, and effectiveness by increasing focus on patient's and provider’s needs [7, 8]. Indeed, HCD involves an iterative, collaborative, and people-centered approach to problem-solving [9] based on the following principles: the active engagement of users, an iterative design process, and the setup of multidisciplinary teamwork. It is being used across diverse industries to solve complex problems, such as redesigning elementary school curricula to improve student engagement [10] and in areas that are high-risk prone, such as aviation [11] and healthcare. *Due to the complexity of the interventions and the critical nature of the decisions, errors can result in serious consequences for patient safety and health outcomes.* Despite this characteristic of healthcare, HCD has already been applied to various situations in health services, with successful results in their improvement. [12–15]

The healthcare industry has acknowledged the significance of HCD and design thinking as pioneering approaches to fulfilling individuals’ requirements and making substantial gains by systematically adopting them. By leveraging human-centered research, diverse and collaborative teamwork, and rapid prototyping, design can foster novel solutions to multifaceted and persistent health issues [6], namely, those where existing practice paradigms do not work and require entirely new approaches to the problem. Moreover, design thinking shares many principles with process improvement commonly used in healthcare management, such as focusing on brainstorming, user requirements, and collaboration. [16]

To achieve it, HCD approaches require that health systems (1) establish the capacity to identify and articulate explicit and implicit stakeholder needs and desires, (2) engage a broader spectrum of voices (particularly those outside of healthcare), and work constructively with divergent perspectives and tensions, and (3) experiment with multiple hypotheses and potential solutions within the target communities.

*Applying HCD principles is particularly relevant in maternity care, given the need for personalized, respectful, and patient-driven clinical and non-clinical (e.g., social, demographic,…) approaches. However, limited studies have focused on its applicability in this area, which supports further research, critically assessing how HCD can advance the humanization of maternity care while identifying future research areas.*

This article critically analyzes the literature by building on the current studies and identifying research gaps to propose fresh avenues for future studies on the interplay between human-centered design and maternity care. It explores whether and how HCD can contribute to the humanization of maternity care, addressing three core questions:

1. Is there a relationship between HCD and the humanization of maternity care?
2. What is the impact of HCD on the humanization of maternity care?
3. What HCD methods and tools can provide more humanized maternity care experiences?

## Methods

To better capture the essence of the interplay between human-centered design and maternity care and address the research questions, a systematic literature review was conducted integrating bibliometric and structured review principles [17].

Studies involving maternity care users (women in the perinatal period and their families) and healthcare professionals in a maternity care setting undergoing any human-centered design approach were included in this review. Quantitative and qualitative study designs, systematic reviews, guidelines, and opinion papers were considered, while textbooks were excluded. The characteristics of the participants (such as age, obstetric risk level, and socioeconomic status) and key intervention elements (design approach, HCD stages, and contexts of implementation) were sought for each study. Subject research areas included Medicine, Nursing, and Multidisciplinary, excluding Pediatrics. All studies that used different design approaches besides experience design, design thinking, user experience, or human-centered design were excluded from this review.

### Search strategy

The search strategy followed a three-step process to identify published and unpublished studies that met the inclusion criteria. Initially, a limited search of MEDLINE (Pubmed), CINAHL (EBSCO), Web of Science, and Scopus was undertaken. These databases were chosen due to their wide coverage of healthcare and design-related disciplines. MEDLINE (PubMed) and CINAHL (EBSCO) are key resources for healthcare literature. At the same time, WoS and Scopus offer a broader range of multidisciplinary content, including design and social science studies, pertinent to HCD in maternity care.

An in-depth search was carried out using search strings adapted to each database. The search terms included a combination of keywords, MeSH terms, and free-text words related to human-centered design (HCD) and maternity care. The search strings were adapted to each database's specific characteristics and indexing rules. The complete search strategies, including Boolean operators, filters, and date restrictions, are shown in Table 1. An example of the search string for MEDLINE (PubMed) is as follows:

- (("Design Thinking" OR "User Experience" OR "Human-Cent* Design") AND (Maternity OR "Maternity Care"))

**Table 1:** Complete search strategy for each database

Database	Search string	Filters applied
MEDLINE (PubMed)	("Design Thinking" OR "User Experience" OR "Human-Cent* Design") AND (Maternity OR "Maternity Care")	Language: English, Spanish, French, Portuguese Year: 2018 onwards Document Type: Articles, reviews
CINAHL (EBSCO)	("Design Thinking" OR "User Experience" OR "Human-Cent* Design") AND (Maternity OR "Maternity Care")	Language: English, Spanish, French, Portuguese Year: 2018 onwards Peer-reviewed: Yes
Web Of Science	("Design Thinking" OR "User Experience" OR "Human-Cent* Design") AND (Maternity OR "Maternity Care")	Language: English, Spanish, French, Portuguese Year: 2018 onwards Document Type: Articles, reviews
Scopus	TITLE-ABS-KEY ("Design Thinking" OR "User Experience" OR "Human-Centered Design") AND ("Maternity" OR "Maternity Care")	Language: English, Spanish, French, Portuguese Year: 2018 onwards Document Type: Articles, reviews

This string was modified for the other databases as necessary, respecting MeSH terms, descriptors, and platform-specific syntax.

Studies published in English, Spanish, French, and Portuguese were considered for inclusion to ensure a diverse representation of global research while considering language constraints.. In addition, studies published from 2018 onwards were included, considering the guidelines published by WHO for a positive childbirth experience [3] and the increased focus on maternal health in recent years, aligning with current global standards. In addition, opinion papers, editorials, and non-empirical studies were excluded, the search restricted to peer-reviewed empirical studies, systematic reviews, and guidelines. In CINAHL, a specific filter for “peer-reviewed” studies was applied.

### Study selection

Following the search, all identified citations were collated and uploaded into Mendeley software, and duplicates were removed. Titles and abstracts were screened for assessment against the inclusion criteria for the review. Relevant sources were fully retrieved, and their citation details were imported into Mendeley software. The full text of selected articles was assessed in detail against the inclusion criteria. Two researchers (with design-related and clinical backgrounds) undertook these steps independently, and disagreements were discussed jointly or with a third party.

Several key variables were collected from the 21 included studies during the data extraction process. These variables included participant characteristics (such as age, obstetric risk, socioeconomic status, and geographical location), intervention characteristics (design approach, context of application, and specific phases of HCD employed), and outcomes (participant experiences, healthcare process changes, and clinical outcomes). For studies where information on participant demographics was missing, it was assumed that the participants met the general criteria outlined in the methods section of the respective studies. This comprehensive approach to variable collection facilitated a comparative analysis of the role and impact of Human-centered Design in maternity care.

During the screening, 127 articles were excluded because they did not fulfill the inclusion criteria. Many studies had an irrelevant focus, addressing neonatal care or unrelated interventions rather than maternity care or human-centered design. Others fell outside the scope of the population, as they did not assess maternal outcomes or target non-maternal populations. Some studies were excluded due to methodological discrepancy, as they were based on alternative conception models rather than human-centered conception principles. In addition, non-empirical sources such as opinion pieces and editorials were not included. Finally, studies published in languages that did not fit the specified criteria or did not include accessible full texts were excluded. This rigorous process ensured that only relevant studies were included in the review.

**Fig. 1:**
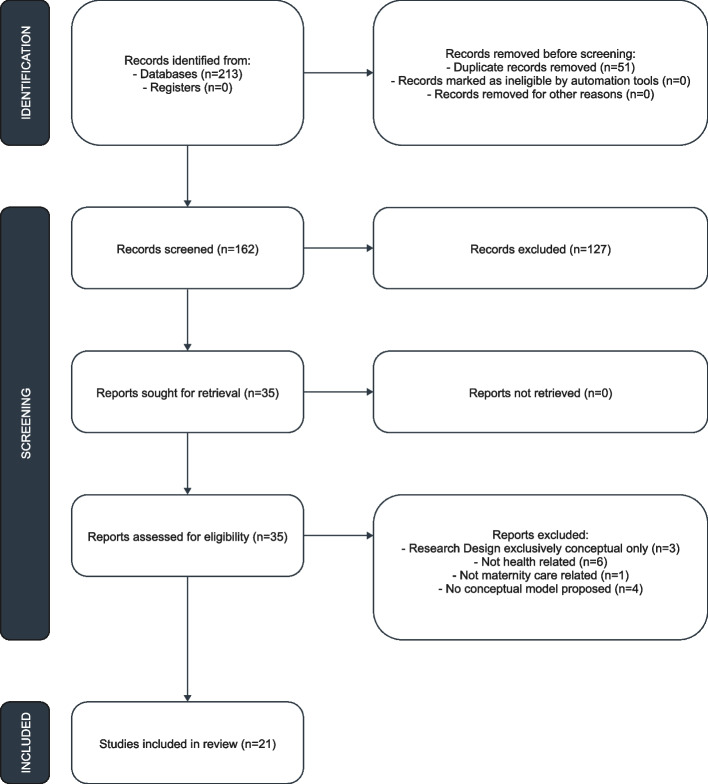
PRISMA flowchart

The research results of the study inclusion process are presented in a Preferred Reporting Items for Systematic Reviews and Meta-analyses extension for scoping review (PRISMA-ScR) flow diagram (Fig. 1).

This review was not registered in any public registry (e.g. PROSPERO), and no formal protocol was drawn up before the study was carried out. Consequently, no amendments to a pre-existing protocol were applicable. Furthermore, this review received no financial or non-financial support, and no external funder or sponsor influenced the design, conduct, interpretation or reporting of the review.

### Definition of clusters

To understand a connection between the studies and to have a macro of bibliometric networks view, all the papers included in the review were extracted from Mendeley as a RIS file and then inserted in the VOSViewer tool. For building the map, terms isolated from both title and abstract fields were selected using the full counting method for a wider spectrum of terms with a minimum of occurrences of a term of four. Clusters were established based on shared conceptual themes, with terms related to HCD (e.g.., user experience, patient’s experience). Aggregated into specific groups. The VOSviewer algorithm grouped these terms into five main clusters, each individually represented using a different color. Clusters were used to understand dominant themes and potential research gaps in HCD applications for maternity care.

### Research term cluster

**Fig. 2:**
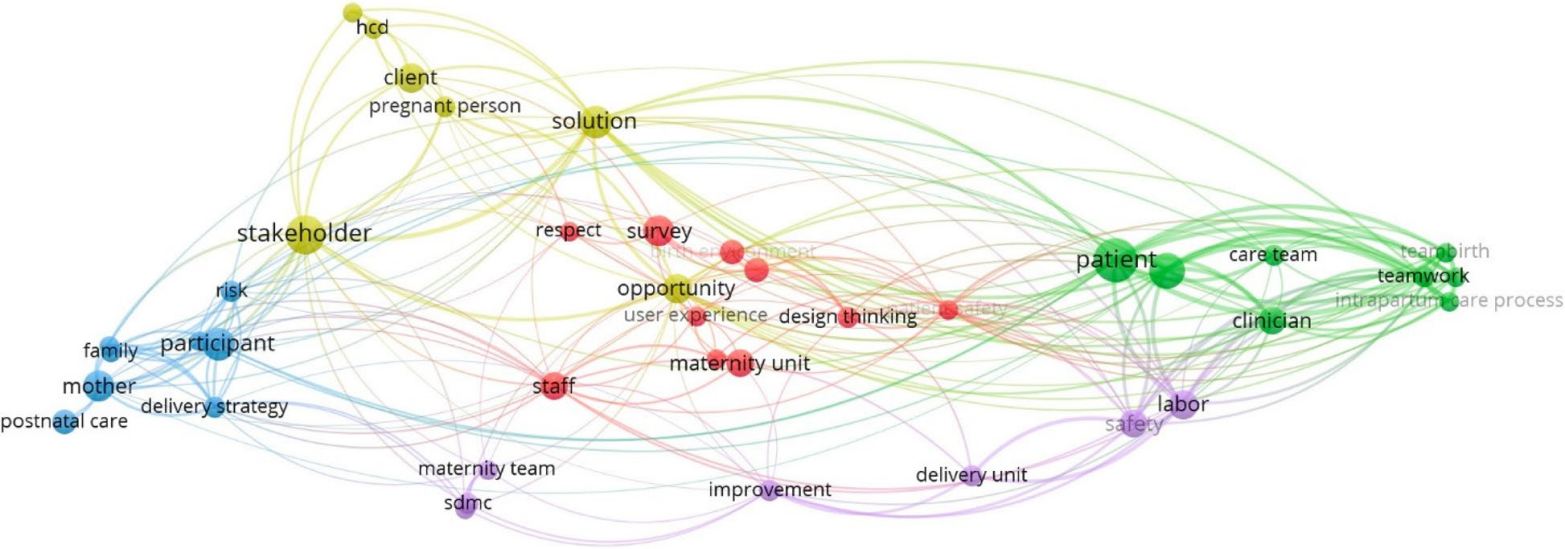
Density view map (via VOSViewer)

VOSviewer software was applied for bibliometric mapping. The results showed five clusters (each identified with a different color), with closely related nodes aggregated within each cluster. The map shown in Fig. 2 was constructed based on the selected data set. The size of each label and circle reflecting the importance of the corresponding term. As observed, terms related to maternity care and health are more recurrent, indicating a strong research focus in these areas. In contrast, terms related to *experience design*, *design thinking*, and *patient/maternal experience* are lesser used or absent, highlighting an opportunity for broader integration of HCD approaches to maternity care.

This visualization represents research density and a practical tool for improving maternity care. By exploring less-dense terms, this density map can identify underexplored areas, revealing new opportunities to incorporate innovative, interdisciplinary methods such as design thinking, which could foster a broader understanding and approach to healthcare services provision. Researchers and institutions can leverage this insight to prioritize emerging themes, guiding the development of collaborative projects that bridge healthcare and design. Moreover, policymakers and funding bodies can use the map to support initiatives targeting these gaps, ensuring that resources are allocated to projects with the potential for meaningful impact on maternal outcomes.

These joint efforts between healthcare professionals, researchers, and design professionals using such visualization maps can trigger the development of user-centered maternity care solutions to provide meaningful maternity care experiences for users and their families. Through this ability to highlight key intersections between different fields of research and science, the density map becomes an essential tool for guiding strategic research planning, shaping policy, and fostering interdisciplinary partnerships that enhance the maternity care landscape globally.

## Results

The sample of twenty-one articles was analyzed, and the results were organized into three tables based on the research questions. Each table presents the relevant studies, their contexts, and key characteristics. This approach allows for better visualization and connection of findings to the specific research questions.

- Question 1: Is there a relationship between HCD and the humanization of maternity care?

### Reasoning for systematizing Table 2

**Table 2:** Studies included in the systematic review, relevant to answer question 1

Author(s)	Article title	Year	Country	Context	Study design	Participants
Afulani P. et al. [26]	Predictors of person-centered maternity care: The role of socioeconomic status, empowerment, and facility type	2018	United States of America	Health	Qualitative	Health professionals and investigators
Avan B. et al. [18]	Inclusive, supportive, and dignified maternity care (SDMC)—Development and feasibility assessment of an intervention package for public health systems: A study protocol	2022	United Kingdom	Health	Mixed-Methods	Postnatal women, healthcare professionals
Bartlett R. et al. [37]	Developing multi-language maternal health education videos for refugee and migrant women in southeast Melbourne	2022	Australia	Health, Education	Qualitative	Migrant pregnant women, healthcare professionals
Filby A. et al. [19]	A service evaluation of a specialist migrant maternity service from the user’s perspective	2020	United Kingdom	Health	Qualitative	Postpartum migrant women
Niles P. et al. [24]	I fought my entire way: Experiences of declining maternity care services in British Columbia	2021	United Kingdom	Health	Qualitative	Pregnant women and healthcare professionals
Nijagal M. et al. [30]	Using human-centered design to identify opportunities for reducing inequities in perinatal care	2021	United States of America	Health, Finance, Education	Mixed-Methods	Low-income pregnant women and partners, healthcare professionals, health-related individuals
Skoko E. et al. [35]	Findings from the Italian babies born better (B3) survey	2018	Italy	Health	Mixed-Methods	Health professionals and investigators

The studies included in this table focus on the conceptual and empirical links between Human-Centered Design (HCD) and humanized maternity care practices. These articles address how HCD principles, such as user-centered approaches and the active engagement of stakeholders, can foster a more personalized and dignified approach to maternity care. For instance, studies in this category examine predictors of person-centered care, explore the perspectives of migrant women in specialized maternity services, and identify inequities in perinatal care. Addressing this question allows to understand the foundational relationship between HCD and the overarching goal of humanized care in maternity settings.

- Question 2: What is the impact of HCD on the humanization of maternity care?

### Reasoning behind systematization of Table 3

**Table 3:** Studies included in the systematic review, relevant to answer question 2

Author(s)	Article title	Year	Country	Context	Study design	Participants
Aggarwal R. et al. [27]	The design of “TeamBirth”: A care process to improve communication and teamwork during labor	2021	United States of America	Health, Education	Mixed-Methods	Healthcare professionals, doulas, health dedicated investigators
Atukunda E.C. et al. [38]	mHealth-Based Health Promotion Intervention to Improve Use of Maternity Care Services Among Women in Rural Southwestern Uganda: Iterative Development Study	2021	Uganda, United States of America	Health	Mixed-Methods	Health professionals and hospital administrators
Breedlove G. et al. [28]	Facility Design: Reimagining Approaches to Childbirth in Hospital and Birth Center Settings	2019	United States of America	Health, Finance, Design	Mixed-Methods	Patients and healthcare professionals
Isangula K. et al. [36]	Strengthening Interpersonal Relationships in Maternal and Child Health Care in Rural Tanzania: Protocol for a Human-Centered Design Intervention	2022	Tanzania	Health	Qualitative	Pregnant women, nurses and midwives, and hospital administrators
McLeish J. et al. [23]	First-Time Mothers’ Expectations and Experiences of Postnatal Care in England	2020	United Kingdom	Health	Qualitative	Pregnant women and healthcare professionals
Salisbury T. et al. [25]	Integrating human-centered design into the development of an intervention to improve the mental well-being of young women in the perinatal period: the Catalyst project	2021	United Kingdom	Health	Qualitative	Young pregnant women, healthcare professionals, government
Sherman J. et al. [31]	Understanding the Heterogeneity of Labor and Delivery Units: Using Design Thinking Methodology to Assess Environmental Factors that Contribute to Safety in Childbirth	2020	United States of America	Health	Qualitative	Pediatricians, obstetricians, nurses, experts in engineering, design, and human factors

The articles in this table explore the practical applications of HCD in maternity care settings and their measurable impacts on care quality and humanization. These studies highlight specific interventions designed with HCD principles, such as improving communication during labor (e.g., the "TeamBirth" care process), promoting mental well-being in perinatal women, and enhancing facility designs to better support patient needs. By analyzing these interventions, the articles provide insights into how HCD-driven solutions can tangibly transform maternity care experiences for both patients and providers. This question focuses on assessing the outcomes and benefits of applying HCD principles in real-world maternity care contexts.

- Question 3: What HCD methods and tools can be used to design meaningful maternity care experiences?

### Reasoning behind systematization of Table 4

**Table 4:** Studies included in the systematic review, relevant to answer question 3

Author(s)	Article title	Year	Country	Context	Study design	Participants
de Mooij M. et al. [29]	OB Nest: Reimagining Low-Risk Prenatal Care	2018	United States of America	Health	Qualitative	Pregnant women and families, healthcare professionals
Edmonds B. et al. [32]	Creation of a decision support tool for expectant parents facing threatened periviable delivery: Application of a user-centered design approach	2019	United States of America	Health, Finance, Education	Mixed-Methods	Pregnant women with periviability labor, pregnant women during periviability, families, and healthcare professionals
Folmer M. et al. [33]	Design of Genuine Birth Environment: Midwives Intuitively Think in Terms of Evidence-Based Design Thinking	2019	Denmark	Health, Design	Qualitative	Patients and healthcare professionals
Geary R. et al. [20]	A step-wise approach to developing indicators to compare the performance of maternity units using hospital administrative data	2018	United Kingdom	Health	Mixed-Methods	Maternity care facilities and hospitals
Grym K. et al. [34]	Feasibility of smart wristbands for continuous monitoring during pregnancy and one month after birth	2019	Finland	Health	Mixed-Methods	Health professionals and hospital administrators
Isaacs R. et al. [21]	Design errors in vital sign charts used in consultant-led maternity units in the United Kingdom	2019	United Kingdom	Health, Design	Quantitative	No participants
Joyce S. et al. [22]	Wait and transfer, curate and prosume: Women’s social experiences of birth spaces architecture	2021	United Kingdom	Health	Mixed-Methods	Pregnant women, healthcare professionals, and designers

This table includes studies that emphasize the specific tools, frameworks, and methodologies of HCD used to enhance maternity care services. Examples include the development of smart wristbands for continuous monitoring, decision support tools for expectant parents, and evidence-based design thinking applied to birth environments. These studies provide practical examples of how HCD methods, such as prototyping, iterative design, and collaborative engagement with diverse stakeholders, contribute to creating meaningful and effective maternity care solutions. This question focuses on identifying and cataloging the actionable strategies and innovations enabled by HCD in maternity care.

#### Demographic characteristics

Most of the studies took place in the United Kingdom (*n* = 8) 18–25, followed by the United States of America (*n* = 7). (26–32) The remaining articles originated from Central Europe (*n* = 3)33–35, Tanzania (*n* = 1)36 and Australia (*n* = 1)0.37 Only one study was multicentric, involving two countries (USA and Uganda).

#### Participants

One study involved pregnant women solely as participants. [19] Whereas the majority of the studies encompassed both healthcare professionals and pregnant women (*n* = 5) [18, 23, 24, 28, 37] some went further and added to their target population either pregnant women’s families (*n* = 3) [30, 32, 33], hospital administrators (*n* = 2) [25, 36] or design experts (*n* = 2) [22, 31] Five studies, interestingly, did not consider patients in their sample. Indeed, their target population consisted of healthcare professionals and health-related individuals (*doulas*, hospital administrators, and health-dedicated investigators). [26, 27, 34, 35, 38] Where participant information was incomplete or unclear, assumptions were made to categorize participant roles on the study’s stated aims and methods.

#### Outcomes

As described in the Methods section, all the studies included were related to maternity care, covering various aspects such as users, professionals, and contexts. The primary outcome of interest was the role and impact of Human Centered Design (HCD) principles in enhancing the humanization of maternity care. This outcome was examined across several dimensions, including the specific stages of the maternity journey that the studies addressed.

Most studies (*n* = 11) took a broad approach to maternity care, exploring its general aspects without focusing on a specific phase. However, eight studies investigated particular stages of the maternity journey, offering a more detailed overview. Among these, six studies focused on childbirth care, including delivery room environments, teamwork, and communication. [21−22,27–28,31,33] In contrast, two focused explicitly on postpartum care, examining women's experiences and the use of interventions during this critical phase. [23, 37]

Outcomes were categorized based on participant feedback. Changes to healthcare were not explicitly stated; they were inferred from the study’s methods and discussion sections.

#### Methodological approaches

The methodological approaches in the research design included qualitative methods in ten studies and quantitative in one study. [21] The remaining articles (*n* = 10) used mixed-methods approaches (qualitative and quantitative). Across these studies, phased design methodologies were consistently employed to structure the research process. Whereas most studies contemplated a three-phased approach (*n* = 12), six other articles proposed a two-phased methodology. Approaches with more than three steps were conducted in two other studies, with five [36] and seven phases [33] (*n* = 2).

Despite the differences in the structuring of the methodological approach, a standard structure emerged in the selected articles. Indeed, phase one often comprised data collection methods such as interviews and/or questionnaires, observational studies (with audio and/or video data), and/or a literature review on the focused theme. Phase two mainly consisted of a descriptive analysis of the collected data in two-phased studies. Phase three typically included roleplay and simulation of prototype testing among stakeholders, with iterative testing and evaluation. Studies with more than three phases further refined the prototypes and incorporated additional rounds of feedback from stakeholders.

In terms of data analysis, the studies analysed used various methodologies, depending on the objectives and design of the study. Thematic analysis was the most commonly used approach for qualitative data, allowing researchers to identify patterns and perceptions in interviews and observation data. Narrative analysis was used in some studies to explore participants' experiences, in particular to understand user-centered maternity care journeys. In the case of studies that used mixed methods, content analysis was often applied to integrate qualitative insights with quantitative results. These analytical methods ensured a solid interpretation of the data in the studies.

The development of solutions for the mainstay problems throughout the design was applied longitudinally to maternity care and specific times of the maternity journey.

#### Maternity care

Regarding the maternity journey, specific attention was put on socioeconomic and ethnicity issues, given that most authors [18, 19, 26, 30, 37, 38] recognized the latter two as leading to reduced or worsened healthcare access. A panoply of solutions was developed, each integrated into the sphere of the country where the study was undertaken. Some of the proposed prototypes included mobilizing human resources by delivering home a “Service Package” (training packages for at-risk moms [18]), creating a “Support Sister” (a reference entity/person to rely on), or development of Community Centers for Pregnancy. [30] Despite the latter, most of the focus on helping at-risk pregnant women was put on the digital through multiple solutions. Proposed prototypes included mobile phone apps to deliver targeted health-related information [38] or to serve as a platform of communication with a healthcare team (text-based communication), allowing for prenatal care from home and consequent empowerment of the mother and preventing low-threshold doctor seeking. [29] Other authors proposed digital delivery of information on maternity care, with perinatal education programs and real-life videos reporting experiences of women with adverse perinatal outcomes. [32] Finally, one author proposed using a wristband to provide the healthcare team with information on maternal health indicators, giving women a sense of being accompanied and diminishing the feeling of neglection. [34]

#### Childbirth care

Childbirth is the cumulus of the maternity journey. Acknowledging that there are failures in the labor ward dynamics, human and non-human, led to a wide range of solutions in some research works. Recognizing that many causes of preventable harm are rooted in flaws of doctor-patient communication and team communication, some authors proposed the creation of a “Huddle board”, to stay in the labor ward, defining the patients and professionals to intervene in each situation and the strategy to follow (next-step approach). [27] Other authors focused on improving hospital charts to make them apparent to all who look at them. Furthermore, spatial architecture is known to influence birth processes, with an impact on safety and outcomes. One author focused on improving birth facilities, often lacking privacy, to achieve more successful birth outcomes. [28, 33]

#### Postpartum care

After childbirth, women often feel neglected at a time when the focus is put on the newborn. Some of the articles prioritize improving their expectations and reality regarding this period, particularly in women with underprivileged backgrounds. [19, 37] Similarly to the proposed prototypes in maternity care, the authors propose the creation of informative media tools to address the postpartum period.

## Discussion

The use of HCD in maternity care remains a phenomenon with little evidence and documentation of how it affects the shape, execution, and outcome of maternal care services. This review reveals that HCD introduces techniques that build empathy to establish emotional and practical links between designers and users (women and healthcare professionals). This facilitates the identification of challenges and the generation of diverse solutions.

Current healthcare systems often need to pay more attention to the human context and needs of stakeholders, leading to unused products or services. Results underscore the importance of incorporating HCD approaches to ensure interventions that resonate with user needs and effectively address systematic gaps, as highlighted by the WHO. WHO’s recommendations emphasize integrating women-centered interventions and outcomes for intrapartum care, including service users’ experiences. This is of particular importance for marginalized groups, such as poor, unemployed, younger, illiterate, unmarried, migrant women, or women from minority groups, for whom person-centered care is very rarely applied or considered. [26] It is essential to note that HCD is not limited to low-risk pregnancies. HCD interventions can enhance communication between women and healthcare providers, allowing collaborative decision-making and improving the overall care experience.

Despite its promise, skepticism persists regarding how effectively innovation, including HCD approaches, can be implemented in healthcare. The time, resources, and effort required remain significant barriers to realizing HCD’s full potential in maternity care settings. Nonetheless, HCD approach provides a promising entry point for strengthening provider–client relationships by inviting clients to co-design acceptable and feasible interventions. [36] Mothers value respectful, skilled, and loving care that gives them a strong sense of personal achievement and confidence, and HCD interventions should support this. [35]

Through HCD, maternity care services can build support tools that combine unbiased information and individualized outcomes with family testimonials to make a meaningful contribution to shared decision-making in maternity care. [32] In cases of High-risk pregnancies, HCD can address specific concerns related to complications, providing individualized solutions that encompass both medical needs and emotional well-being. This approach can help ensure that care remains respectful and compassionate, even when complex medical interventions are necessary.

To achieve this, it is recommended that facilities employ a comprehensive training package (on respectful and supportive maternity care) and a HCD strategy to ensure the implementation of recommended practices around supportive and dignified care in routine, facility-based maternity care. [18] The use of patient-centered care models within the HCD framework can transform maternity care by providing a more desirable, participatory experience for expectant mothers and their care teams. By stratifying pregnancies according to risk and support needs, the aim is to demedicalize the pregnancy experience by providing a supportive and empowering experience that fits within the women’s daily lives. Using an iterative HCD approach, a low-risk perinatal care model can be implemented, which reduces the overall need for in-clinic visits and engagement of an entire care team, offering the potential for a lower-cost alternative to traditional care. [29]

Even though a total of three models were mentioned in some of the studies, IDEO, a global design firm, was the one that popularized human-centered design, narrowing it down into three phases: (1) inspiration, (2) ideation, and (3) implementation. [39] The inspiration phase is about learning from the people you are designing for. To develop a good solution, it is essential to discover what the user wants firsthand rather than build products or services based on preconceived ideas about what you believe they want. This stage requires the ability to comprehend customers’ experiences and emotions – empathy. [9] Different research methods can help better understand the people you are designing for, such as interviews, guided tours, observations, immersions, and secondary research. [40] In the ideation phase, the main goal is to brainstorm as many ideas as possible, integrating the feedback from the previous step. Collecting insights, testing, and iterating on those ideas is essential until you reach an ideal solution. There are various tools to choose a solution to take forward, namely, journey maps, co-creation sessions, business model canvas, and storyboards. Finally, during the implementation phase, the objective is to bring the chosen solution to the market based on the user’s location and how they prefer to be marketed. Given that the customers’ wants and needs will continue progressing, the iteration should always continue. The following methods are examples of what can be used to assess if the solution is working and prepare it for launch and scale: life prototyping, a roadmap for success, monitoring, and evaluation, and creating a pitch.

Across the studies reviewed, the concept of HCD was applied in diverse ways. Several studies emphasized the role of empathy in understanding user need, whether through in-depth interviews, co-creation workshops, or interactive prototyping sessions centered on the feedback of pregnant women and healthcare professionals [9, 18, 25, 26]. Others highlighted the implementation phase of HCD, where user-informed solutions were transformed into ºpractical tools, such as mobile health applications, community support platforms, or special redesigns of maternity wards [29, 31, 32, 37]. These approaches underline how HCD gathers insights and actively integrates user input into the design and deployment of maternity care solutions. Furthermore, the reviewed papers demonstrated how HCD frameworks contribute to healthcare systems reforms by prioritizing patient and stakeholder feedback to ensure interventions resonate with those they aim to serve.

The two remaining models consist of a five-phase design process. The first one includes (1) empathizing with stakeholders, (2) defining the problem, (3) generating ideas for solutions, (4) prototyping the solutions, and (5) testing the solutions. On the other hand, the second one includes (1) understanding and specifying the context of use, (2) defining customer requirements, (3) creating design solutions, (4) evaluating design against requirements, and (5) delivering design solutions that meet user conditions. [9]

Labor and childbirth are a particularly vulnerable time for women, and it is but natural that women attach great value to the care they receive during this time. Their satisfaction hinges upon quality care that achieves or surpasses their expectations. However, healthcare systems continue to rely on a limited definition of quality based solely on clinical metrics. This proves an emphasis on biomedical measures over psychosocial experiences and demonstrates the gap between what women value and what the system values. [24] HCD fills this gap by expanding the focus beyond clinical outcomes to encompass holistic care experiences [9, 29, 31, 32]. By integrating women's lived experiences, emotions and specific needs, HCD offers a framework that values not only health outcomes, but also the qualitative dimensions of care, such as respect, autonomy and trust, which are often neglected in biomedical models [3, 18, 36]. This review’s findings have revealed that women’s care experience is affected by various determinants that could influence their future care utilization. Migrant women [19] and women in the postpartum period [23] are particularly dissatisfied with the care they routinely receive. Maternal health programs and policies must consider women’s perspectives on the care they need and their feedback on the services they receive.

HCD can provide a robust system of obtaining client feedback and utilizing such information to improve services. Performance and experience indicators are developed collaboratively in ways that matter to patients. For an indicator to be considered valid, it must also be likely that a difference in the indicator reflects a difference in the quality of care, and a specific direction should reflect better quality. [20]

Telehealth tools, including smartphone apps [29, 32], text messaging plattforms [29, 38], moderated online communities [29], electronic self-monitoring tools [29, 34] and virtual reality tools [32], improve timely communication of health-related information and produce positive health behavior change and health outcomes. It is also recommended to create informative media tools, using a design thinking methodology, to address relevant information regarding prenatal, antenatal, and postnatal care to specific target groups. [37] Furthermore, design thinking can be implemented at various stages of healthcare facility building projects to improve hospital systems and environmental factors. In most hospitals, labor and delivery units are heterogeneous in design, needing more consistency regarding environmental factors that may impact safety and outcomes. Most building codes must consider workflow, human factors, and patient and clinician experience. However, it is known that physical surroundings can affect the performance of staff and the mother’s perception and experience satisfaction. A first step to improving maternity care is improving birth settings using HCD in spatial architecture, staffing, and policy development. [22, 28] Examples of areas actionable through design are appropriate neonatal resuscitation spaces [31], supply and equipment stocking and organization methods [31], blood banks accessibility [31], and observation charts functional design. [21]

Despite these advancements, this review found no direct evidence linking HCD methodologies to improvements in maternal morbidity and/or mortality, aside from perinatal mental health. Most mothers often report feeling unsupported during pregnancy and postpartum, especially regarding mental health and perinatal education. HCD interventions should focus on providing women with perinatal information that improves their health and emotional well-being, strengthens their communication and problem-solving skills, and fosters community support networks. [25] Specifically, in situations of pre-viability births, interventions should be individually tailored to support parents in grief and bereavement. [32] In this sample, no articles addressed obstetric outcomes, namely birth interventions (labor inductions, cesareans, instrumented births, episiotomies), fetal mortality/morbidity rates, or other related outcomes (ex: breastfeeding). Nevertheless, though indirectly related, one article focused on the applicability of design processes to teamwork in labor ward to improve outcomes. [27]

### Limitations of HCD implementation

The application of HCD to healthcare still needs to be developed. One example is the need to provide integrated HCD approaches and methods to develop health innovations. [9] Also, final testing and implementation of the projects should be present in most of the studies in question. In summary, only some studies discuss the interplay between HCD and maternity health care, and there is still a long way to go in this field.

### Limitations to the review

Despite the particular scope of this review, some limitations should be considered. Firstly, the application of HCD to healthcare still requires further development, with a need to integrate approaches and methods more consistently in the development of healthcare innovations. In addition, the lack of final tests and prolonged implementation of the interventions proposed in most of the studies makes it difficult to assess the lasting impact of the solutions robustly. Finally, little research has systematically addressed the interrelationship between HCD and maternity care, highlighting a field yet to be explored and suggesting a long way to go before HCD is fully incorporated into clinical practice and maternity services. These limitations reinforce the need for future, more standardized studies that include rigorous methodological evaluations and long-term impact analyses.

## Conclusion

HCD approaches are increasingly helpful tools in surpassing systems’ problems and disadvantages, gaining relevance in different areas of healthcare. Their growing applicability in healthcare systems might become a relevant way of identifying and targeting healthcare systems’ gaps, such as the ones lacking resolution already identified by the World Health Organization regarding Maternity Care worldwide.

HCD provides healthcare systems with a practical, transformative framework for responding effectively to gaps and aligning interventions with the needs of all stakeholders. Its integration into maternity care could address significant unresolved issues while paving the way for improved experiences and outcomes. This includes low-risk and high-risk pregnancies, its principles can be applied to improve care for women facing complications, ensuring that care is both compassionate and clinically sound.

This review identified several global on HCD approaches to improve the maternal and childbirth journey. While emerging in literature worldwide, their application to maternity care is still being determined. Future empirical studies evaluating human-centered design's applicability in maternity care are needed, particularly to understand what drives the experience of women and their relatives.

WHO and other international organizations support applying design thinking in healthcare as a pioneering strategy to fulfil women’s needs and improve maternal outcomes. Systematic adoption of such approaches offers opportunities to bridge critical gaps in maternity care while improving experiences for women and families. This aligns with WHO’s call to address social, demographic, and migratory issues in healthcare. Although some countries report improvements in childbirth morbimortality rates, studies show plenty of room to improve maternity experience touchpoints during labor and birth care, with reporting evidence that such improvements potentially impact mothers’ willingness to have future babies. By applying HCD to both low-risk pregnancies, healthcare systems can bridge these gaps, fostering environments that are supportive and responsive to the diverse needs of all women. This includes addressing the unique emotional and physical challenges faced by women in high-risk pregnancies.

As former UN Secretary-General Ban Ki-Moon emphasized, “Put women at the center of care, enhancing their experience of pregnancy and ensuring that babies have the best possible start in life.” To achieve this vision, healthcare professionals must allocate more resources to research and healthcare. These investments will empower care teams to meet women's diverse and individualized needs in the perinatal period, laying a strong foundation for deploying HCD in maternity care.

## Data Availability

No datasets were generated or analysed during the current study.
